# Considerations for the Successful Detection and Quantification of Genetically Modified Events in Grain and Food Samples Using Multiplex Digital PCR

**DOI:** 10.3390/foods14010075

**Published:** 2024-12-31

**Authors:** Tigst Demeke

**Affiliations:** Grain Research Laboratory, Canadian Grain Commission, Winnipeg, MB R3P1N1, Canada; tigst.demeke@grainscanada.gc.ca

**Keywords:** digital PCR, GMO detection, optimization, multiplex dPCR

## Abstract

The number of genetically modified (GMO) events for canola, corn, and soybean is steadily increasing. Some countries, including those in the EU, have regulatory requirements for the approval and use of plant ingredients containing GMOs. Multiplex digital PCR (dPCR) has been used for the simultaneous detection and quantification of various GMO events. This review covers the various factors to consider for multiplex digital PCR detection and the quantification of GMO events. DNA quality, quantity, and the presence of inhibitors are important factors to consider. Some dPCR instruments allow the use of multiple fluorescent dyes, which facilitates the setup of multiplex dPCR assays. This review focuses on the optimization of multiplex dPCR and describes the multiplex dPCR assays that have been reported for GMO detection.

## 1. Introduction

The number of genetically modified (GM) events for canola, soybean, and corn has been steadily increasing, necessitating the development of efficient detection methods to meet regulatory requirements in many countries (https://www.isaaa.org/gmapprovaldatabase/default.asp, accessed on 28 December 2024). Digital PCR (dPCR) is being widely used for the detection and quantification of genetically modified (GM) events. Unlike real-time PCR, dPCR does not require reference materials or standard curves, making detection and quantification relatively easier [1,2]. Digital PCR inherently reduces the impact of PCR amplification efficiency variations, resulting in improved precision in quantification, especially for low-abundance targets (https://www.qiagen.com/us/knowledge-and-support/knowledge-hub/bench-guide/pcr/digital-pcr/what-is-digital-pcr, accessed on 28 December 2024). Partitioning is performed in prefabricated chambers using nanofluidics or by generating droplets of water-in-oil emulsion [3]. The reaction mixture in each partition undergoes PCR amplification using fluorescence-based detection. Once the PCR is completed, the partitions are examined to determine which ones contain amplified product for each target. Quantification is performed by counting the number of positive partitions for each target, which will help to determine the absolute concentration of each target. Quantification is based on Poisson statistics, as the partitioning creates a discrete digital distribution of target molecules. A Poisson model is used to determine the probability of a microreaction receiving zero, one, two, or more copies of the target molecule (https://www.qiagen.com/us/knowledge-and-support/knowledge-hub/bench-guide/pcr/digital-pcr/what-is-digital-pcr, accessed on 28 December 2024). Poisson statistics allows for the calculation of the initial number of targets from the number of positive and negative partitions.

Multiplex dPCR enables the simultaneous detection and quantification of two or more target nucleic acid sequences in a single reaction, enhancing efficiency and conserving resources. Many of the dPCR instruments have a limited number of fluorescence detection systems. For example, the commonly used dPCR instruments such as BioRad QX200 have two fluorescent channels. The optimization of the dPCR is important to ensure the correct detection and quantification of two or more DNA targets in multiplex setups. There are various strategies to achieve multiplexing in dPCR, one of which involves adjusting the primer and/or probe concentrations used in the reaction. The various approaches for achieving multiplexing in dPCR are described below. Several factors influence the success of both single and multiplex dPCR assays, and these critical factors are discussed at the beginning of the review chapter.

## 2. Factors to Consider for Optimization of Digital PCR (dPCR) Assays

### 2.1. Effects of DNA Quality and Inhibitors on dPCR

Good-quality DNA suitable for PCR can be extracted using traditional methods, such as CTAB (cetyltrimethylammonium bromide) and SDS (sodium dodecyl sulphate) techniques [4,5]. Further purification of the extracted DNA may be needed to reduce inhibition. The CTAB and SDS methods are generally time-consuming and can also use inorganic chemicals such as phenol/chloroform. Commercially available kits are preferred as they are fast and some of them do not use hazardous chemicals. However, not all test kits are suitable for extracting DNA from different grains or food ingredients [6]. The amount and quality of DNA extracted from canola, flax, and soybean seeds varied widely. DNA extracted with the CTAB method and purified with the DNA Clean & Concentrator kit was suitable for both dPCR and real-time PCR analysis of GE canola, flax, and soybean events. Flax DNA, extracted with many of the kits, was viscous and the clear distinction of target clusters was not achieved for dPCR. It is important to verify the suitability of extracted DNA for PCR. The DNA extraction method for maize seed and grains has been validated by the European Reference Laboratory for GMO food and feed (https://gmo-crl.jrc.ec.europa.eu/summaries/CRL-VL-16-05-XP-Corrected-version-2.pdf, accessed on 28 December 2024). The accurate determination of DNA concentration in the extracted samples is necessary. Fluorometric or spectrophotometric methods can be used to determine DNA concentrations [4,7].

The quality of the genomic DNA used affects PCR results. Inhibitors in the DNA include organic compounds such as phenol, sodium dodecyl sulphate (SDS), polysaccharides, and proteins [8]. Acidic polysaccharides such as dextran sulphate are inhibitory to the PCR [9]. Some of the inhibitors such as proteases or detergents can degrade DNA polymerase [8]. Digital PCR has been reported to be less sensitive to inhibitors in the DNA [1,10]. Droplet digital PCR (ddPCR) was less affected by SDS compared to quantitative real-time PCR. Many PCR inhibitors target the DNA polymerase directly or indirectly. According to Dingle et al. [11], the ddPCR assay was more tolerant to SDS and heparin than the qPCR assay. The success of the ddPCR assay was attributed to reaction partitioning through digitization, which may have resulted in reduced susceptibility. For sample or target combinations with low levels of nucleic acids and/or variable amounts of chemical and protein contaminants, ddPCR technology produced more precise and reproducible results [12]. However, dPCR can be affected by the presence of inhibitors in some cases, and so checking the suitability of the extracted DNA is helpful [6].

The presence or absence of PCR inhibitors can be verified by testing different dilutions prepared from the DNA solution [7]. Two or more DNA dilution levels can be tested by PCR using a taxon-specific reference gene. The threshold (Ct) value from the more concentrated DNA solution is compared to the Ct values of the less concentrated DNA solutions and to the expected value in the absence of inhibitors [7]. Generally, the effects of inhibitors can be reduced by selecting an appropriate method for sample processing and nucleic acid extraction, choosing a more robust DNA polymerase, or using specific PCR additives [6,7].

### 2.2. Impact of DNA Degradation on dPCR

The quality and integrity of DNA affects PCR amplification. The type of sample (e.g., processed sample) and the DNA extraction method used can result in DNA degradation. The underestimation of GM content has been reported because of DNA degradation [13]. The use of high temperatures or pressure for the treatment of flour samples significantly reduced the level of detectable GM soybean and maize DNA [14]. For amplicons of <200 bp, there was amplification with qualitative PCR. DNA sequences with high GC content were more heat-stable compared to the ones with low GC contents [14].

In other studies, DNA degradation was reported to a have minimal effect on real-time quantitative PCR results [15,16]. Plasmid DNA samples were subjected to heating, baking, microwaving, autoclaving, and ultraviolet irradiation to determine the effect on real-time PCR results [16]. The transgenic and taxon-specific reference DNA sequences were equally affected by the treatments and the PCR result was not affected. This has been shown to be the case when short amplicons of similar sizes are used for both the target and taxon-specific DNA sequences. The use of DNA extraction methods optimized for specific food matrices is supposed to improve GMO detection in processed foods [17].

Ground samples spiked at 0.1% and 1% GM content were heat-treated in boiling water for 15, 30, and 60 min and DNA was extracted from the treated as well as non-treated samples [18]. The amount of DNA obtained from degraded samples was much lower than that obtained from non-degraded samples. Both digital PCR and real-time quantitative PCR results were not affected as a result of DNA degradation for three of the four GMO events tested. For one event, a higher GMO content was observed for the 0.1% and 1% concentrations used. DNA degradation will have a higher impact on PCR amplicons of large sizes. The amplification of small amplicon sizes (≤100 bp) for both target and reference DNA sequences appears to be less impacted by DNA degradation [16].

### 2.3. Effect of DNA Quantity on dPCR

The accurate quantification of GM materials at concentrations below 0.1% is challenging. The quantification of GMOs at low concentration levels is important for the detection of unapproved GMO events. For example, the unapproved FP967 GMO flax (also known as CDC Triffid) was discovered at low concentrations (between 0.01% and 0.1%) in 2009 (https://pmc.ncbi.nlm.nih.gov/articles/PMC4392022/, accessed on 28 December 2024). In most cases, the amount of DNA used for dPCR is similar to that used for real-time quantitative PCR. For real-time PCR, the use of high amounts of DNA was helpful for the quantification of low amounts of GM materials [19]. The detection of a 0.005% GM maize sample was achieved by the use of 500 ng DNA rather than the use of 50 ng DNA. DirectAce qPCR master mix, which has high tolerance to PCR inhibitors, was used for the experiment [19]. The effect of varying DNA amounts on the detection of low levels of GM materials was investigated using dPCR [20]. Digital PCR provides high precision at both low and high DNA concentrations compared with real-time PCR, but very high DNA concentrations can saturate the system, reducing the dynamic range. The use of 400 and 600 ng DNA compared to 50 and 100 ng DNA improved the quantification of GM canola and soybean events at 0.01%. There was a saturation problem with the use of 400 and 600 ng DNA for GM canola events. An excess number of reference droplets were generated at high DNA concentrations, while no negative droplets were formed. Without negative droplets, Poisson correction cannot be applied, making it impossible to calculate the concentration. However, the saturation problem was overcome by the use of multiple wells comprising 200 ng DNA instead of using 400 and 600 ng DNA per well and by pooling the number of droplets [20]. Overall, it is important to determine the optimum amount of DNA to use for dPCR, especially if the target DNA concentration to be determined is at a very low level.

### 2.4. Impact of Taxon-Specific Reference Gene Choice on dPCR Results

The type of reference gene used for dPCR affects the quantification of GMOs [21,22]. This is because of the fact that dPCR results are determined in relation to taxon-specific reference genes. A taxon-specific assay should be specific to the crop of interest, consistent and stable across different varieties, and ideally target a single-copy gene in the plant genome to ensure accurate and reliable detection and quantification [22]. The most commonly used taxon-specific real-time PCR assays, validated by the European Union Reference Laboratory for Genetically Modified Food and Feed, were evaluated for their suitability in dPCR. Single-copy target taxon-specific reference genes were identified for canola, soybean, corn, and cotton [22]. Regarding the ddPCR quantification of GMOs, *lec A* and *lec B* are suitable for soybean; *hmg* and *ZmAdh1* are suitable for maize; *FatA(A)* is suitable for oilseed rape; and *AdhC* or *SAH7* is suitable for cotton. The expected percentage values were obtained when *FatA(A)* and *HMG-I/Y* single-copy reference genes were used for canola. When a two-copy reference gene (e.g., *Cruciferin*) was used for canola ddPCR, the GM percentage value was underestimated because of the high copy number [21]. However, both single- and two-copy canola reference genes provided the expected GMO percentage values for real-time quantitative PCR assay. Therefore, the use of a single-copy reference gene is crucial for the accurate determination of GMO content in grain and processed samples using dPCR.

### 2.5. Digital PCR Instruments and Droplet Volumes

Various types of dPCR instruments have been developed [23]. The different types of dPCR instruments available are listed in Table 1. The dPCR instruments are either droplet-based or chip-based. Droplet-based methods use water-in-oil emulsions, while chip-based methods use nanofludics to load the reactions in prefabricated chambers or solid-state partitions [2,3]. The DNA targets are amplified with PCR and dedicated readers measure the end-point fluorescence. The use of multiple fluorescent dyes for dPCR instruments will help with the detection of different targets at the same time. For the Bio-Rad QX600, Clarity Plus dPCR, and Naica Stilla instruments, six fluorescent dyes can be used.

Droplet volume variability has also been suggested as a critical factor for absolute quantification using ddPCR [24,25]. Some of the ddPCR instruments were reported to produce lower droplet volumes than stated by the manufacturers. Droplet and partition volume affect the accuracy and uncertainty of dPCR measurements. The droplet volume was also affected by the type of supermix and the type of droplet generator used [25]. Correction-factor-based measurement for two dPCR platforms helped to obtain similar values. Accurately measuring droplet volume for dPCR is challenging for many laboratories. A ratio of transgene to the endogenous DNA copy number is used to determine GMO content and slight variation in droplet volume may not be critical. In general, dPCR methods have been successfully validated without the need for droplet volume adjustments.

## 3. Multiplex Digital PCR for Detection and Quantification of GMOs

In multiplex dPCR, different sets of primers and probes designed for specific DNA sequences of the target genes are incorporated into the same reaction mixture. The technique allows for the simultaneous detection and quantification of two or more GMO events. Multiplex dPCR saves time and resources and provides absolute quantification without the need for a standard curve, making it accurate for the quantification of target DNA sequences. The implementation of a single-target dPCR can be performed quickly as validated real-time quantitative PCR methods can be transferred to a dPCR format (https://op.europa.eu/en/publication-detail/-/publication/fbc6d552-75f5-11e9-9f05-01aa75ed71a1/language-en, accessed on 28 December 2024). The implementation of multiplex digital PCR (dPCR) requires a thorough verification process, which may take more time depending on the number of GMO events to be multiplexed and the dPCR instrument utilized.

### 3.1. Optimization of Multiplex dPCR

The term ‘higher-order multiplexing’ is used to refer to multiplexing that can detect more targets than there are fluorescent detection channels [26]. It is helpful to follow the minimum information guidelines for the publication of quantitative digital PCR experiments [26]. Amplitude-based, ratio-based, and non-discriminating multiplexing methods have been described [3,27]. Primer and/or probe concentrations are varied for use in amplitude-based multiplexing. The difference in probe concentration will result in varying fluorescence amplitudes, allowing the two probes to be distinguished. Higher primer and probe concentrations are supposed to enhance the intensity of the end-point fluorescence signal, which helps to separate the background signal from the target signal [2]. Evaluating primer and probe interactions using in silico tools will assist in the optimization of multiplex ddPCR. Setting up optimal annealing/extension temperatures for the multiplex dPCR assays simultaneously is also helpful. Ratio-based multiplexing involves the use of varying ratios of two probes labeled with different fluorophores to detect multiple targets within a single optical channel [28]. The system allows the detection of more targets than the number of available fluorescence channels. The careful selection of fluorescent dyes is important to minimize spectral overlap and ensure the differentiation of signals. By varying the concentrations of different fluorogenic probes of the same color, it is possible to distinguish between the probes based on fluorescence intensity [29]. For example, a tetraplex ddPCR assay was developed using a 1.5× target 1 probe (FAM), a 1× target 2 probe (FAM), a 1× target 3 probe (VIC), and a 0.6× target 4 probe (VIC) via QX200 (https://www.bio-rad.com/webroot/web/pdf/lsr/literature/Bulletin_6451.pdf, accessed on 28 December 2024).

Non-discriminating multiplexing involves the detection of multiple targets as a group within a single fluorescence channel without differentiating between individual targets [30].

Target linkage, probe specificity, and competition, as well as differential PCR efficiencies, are some of the important factors to consider for the accurate quantification of multiplex dPCR assays [3]. Target linkage can occur as a result of the co-localization of tandem repeat copies of the same target, which leads to the underestimation of copy numbers [3]. The random and independent distribution of target molecules (based on Poisson statistics) may not hold for linked targets. Proper calibration is helpful to account for variations in signal intensity and to correct for bleed-through (crosstalk) between channels. Fluorescent bleed-through occurs when a signal from one fluorophore is detected in a channel intended for another [26]. Methods including reducing the amount of the relevant probe, changing the fluorophore, and/or applying a color compensation matrix have been suggested to minimize bleed-through. Clear separation is essential in fluorescence amplitude between negative and positive targets [31]. When the amplification/hybridization efficiency is sub-optimal, partitions that have intermediate fluorescence values between positive and negative populations are created (also called rain). Methods like the optimization of dPCR, including hybridization temperature, the sonication of DNA, the use of PCR enhancers, etc., are suggested to help reduce rain [32].

The use of ddPCR Multiplex Supermix (e.g., BioRad reaction mix) facilitates the establishment of multiplex dPCR assays by providing optimized reagent conditions that enhance the performance of multiple primers and probes within a single reaction.

Digital PCR instrument-specific software, such as QIAcuity, Bio-Rad QX, QuantStudio Absolute Q, and Naica System, is available for data acquisition and analysis. An R package, ddPCRclust, has been developed for the automated analysis of data from Bio-Rad’s droplet digital PCR systems (QX100 and QX200) [33]. It can automatically analyse and visualise multiplexed ddPCR experiments with up to four targets per reaction. The analysis and visualization of dPCR data in R is also available (https://cran.r-project.org/web/packages/ddpcr/vignettes/overview.html, accessed on 28 December 2024). A universal R-package and shiny app called Cluster Predictor has been developed for the automated analysis of up to 4-plex dPCR data [34].

Carrying out validation experiments with known concentrations of each target helps to ensure that the multiplex dPCR provides accurate and consistent results. The verification of multiplex dPCR methods, in terms of sensitivity, trueness, precision, dynamic range, linearity, and robustness, using known reference materials is important [35]. It is also important to determine the limit of detection (LOD) and the limit of quantification (LOQ) for the multiplex dPCR.

### 3.2. Multiplex dPCR Assays

Duplex dPCR consisting of a target GMO event and a reference gene has been successfully used for the detection and quantification of many GMO events [36,37]. Quantification will be more accurate since the same reaction is used, unlike the separate reactions required for real-time quantitative PCR assays.

Multiplex dPCR procedures have been developed for the detection and quantification of maize, soybean, and canola GMO events. For maize, multiplex dPCR procedures have been reported by different laboratories [38,39,40]. Duplex, tetraplex, and pentaplex assays were successfully used for the detection/quantification of maize GMO events (Table 2). For maize, less biased GMO quantification using two genetic element-specific multiplex dPCR assays was reported [41]. A single universal primer-multiplex-ddPCR (SUP-M-ddPCR) strategy consisting of four genetic elements and one reference gene (35S, NOS, NPTII, PAT and adh1) was reported for the accurate broad-spectrum detection of maize GMO events [40].

Multiplex dPCR procedures, ranging from duplex to hexaplex, have been developed for soybean GMO detection and quantification [36,42,43]. The quantification of five soybean GMO events in a single reaction was performed using Naica six-color Crystal dPCR [43]. Multiplex dPCR consisting of three and four GMO events has also been reported for soybean [36]. A combination of tetraplex element-specific and tetraplex event-specific multiplex ddPCR assays was used to detect 19 soybean GMO events [44]. The element-specific tetraplex ddPCR assay, consisting of P35S, T-nos, Pat and tE9, detected 15 soybean GMO events. The tetraplex ddPCR assay has been reported for the detection and quantification of four canola GMO events [36].

**Table 2:** Examples of multiplex dPCR assays developed for different crops.

Multiplex dPCR				
Crop	Assay Used	dPCR Instrument Used	Reference	Comment
Corn	2-plex	QuantStudio 3D. ThermoFisher (Waltham, MA, USA)	[41]	Element-specific
	4-plex & 10-plex	BioRad QX100. BioRad (Hercules, CA, USA)	[38]	
	4-plex	BioRad QX200. BioRad (Hercules, CA, USA)	[39]	
	5-plex	BioRad QX100	[40]	Universal primer multiplex
Soybean	2 & 3-plex	BioRad QX200	[36]	
	4-plex	BioRad QX200	[44]	Element & event-specific
	4-plex & 6-plex	Naica Crystal dPCR. STILLA (Boston, MA, USA).	[43]	
Rice	3-plex	BioRad QX200	[45]	One universal LNA probe
Canola	4-plex	BioRad QX200	[36]	Event-specific

A universal locked nucleic acid (LNA) probe-mediated dPCR (ULNA) has also been developed for the quantification of multiple DNA targets in rice [45]. In ULNA-ddPCR, only one universal LNA probe is used for multiplex DNA targets.

In most of the cited research, a two-fluorescent-color dPCR system was used for the detection and quantification of more than two GMO events via optimization. The availability of various fluorophores for a given dPCR instrument will aid in establishing multiplex reactions. For example, the BioRad QX600, Clarity Plus and Naica Stilla dPCR instruments have 6 fluorescent dyes (Table 1).

## 4. Summary

Digital PCR is widely used for the detection and quantification of GMOs. The number of GMO events has been steadily increasing and efficient methods are needed to detect and quantify two or more GMO events at the same time. Factors to consider for the optimization of multiplex dPCR assays include DNA quality and quantity, the use of single-copy reference genes, varying primer/probe concentrations, checking fluorescence compatibility/ratio, the choice of digital PCR instruments, the use of dPCR multiplex supermix, and use of optimized dPCR reaction conditions. Optimizing primer and probe concentrations allows the detection and quantification of more than two GMO events using two fluorescent dyes. The availability of various fluorescent dyes (e.g., six dyes for BioRad 600, Clarity Plus and Naica system) in dPCR instruments will enhance the development of multiplex dPCR assays. The validation of the developed multiplex dPCR assay is important to ensure accuracy, reliability, and reproducibility. Multiplex dPCR has been utilized to detect and quantify multiple GMO events in corn, soybean, rice and canola. Successful multiplex dPCR will enhance the acceptability and use of dPCR method for detecting and quantifying GMOs.

## Figures and Tables

**Table 1:** Examples of digital PCR instruments.

Company	Name of Instrument	# Color Detection	Comments
BioRad (Hercules, CA, USA)	QX100	2	Droplet-based
	QX200	2	Droplet-based
	QX600	6	Droplet-based
ThermoFisher (Waltham, MA, USA)	QuantStudio 12k	2	Microchamber-based (OpenArray)
	QunatStudio Absolute		
	Q Digital PCR system	4	Microchamber-based
RainDance Technologies (Billerica, MA, USA)	RainDrop		Droplet-based; *discontinued*
Fluidigm (South San Francisco, CA, USA)	BioMark HD	Up to 5	Chip-based
STILLA (Boston, MA, USA)	Naica system	3	Droplet-based (Crystal dPCR)
	Naica system	6	Droplet-based (Crystal dPCR)
JN Medsys (Singapore)	Clarity Plus dPCR	6	Chip-based. 45K partitions. Chip-in-a-tube
QIAGEN (Hilden, Germany)	QIAcuity Digital	2, 5	Array-based
Formulatrix (Bedford, MA, USA)	Constellation	5–8	Microporous plate technology
OPTOLANE (Yongin-si, Republic of Korea)	Lab on an array	2	Array-based. Real-time PCR & dPCR

Sources: references [23,24]; Company websites and literature search. Comparison of digital PCR methods.

## Data Availability

No new data were created or analyzed in this study. Data sharing is not applicable to this article.
